# Characterization and Application of the Sugar Transporter Zmo0293 from *Zymomonas mobilis*

**DOI:** 10.3390/ijms24065888

**Published:** 2023-03-20

**Authors:** Kun Zhang, Wenwen Zhang, Mengxing Qin, Yi Li, Hailei Wang

**Affiliations:** Henan Province Engineering Laboratory for Bioconversion Technology of Functional Microbes, College of Life Sciences, Henan Normal University, Xinxiang 453007, China

**Keywords:** *Zymomonas mobilis*, sugar transporters, glucose metabolism, bioethanol, high concentrations of glucose

## Abstract

*Zymomonas mobilis* is a natural ethanologen with many desirable characteristics, which makes it an ideal industrial microbial biocatalyst for the commercial production of desirable bioproducts. Sugar transporters are responsible for the import of substrate sugars and the conversion of ethanol and other products. Glucose-facilitated diffusion protein Glf is responsible for facilitating the diffusion of glucose uptake in *Z. mobilis*. However, another sugar transporter-encoded gene, *ZMO0293*, is poorly characterized. We employed gene deletion and heterologous expression mediated by the CRISPR/Cas method to investigate the role of *ZMO0293*. The results showed that deletion of the *ZMO0293* gene slowed growth and reduced ethanol production and the activities of key enzymes involved in glucose metabolism in the presence of high concentrations of glucose. Moreover, *ZMO0293* deletion caused different transcriptional changes in some genes of the Entner Doudoroff (ED) pathway in the ZM4-Δ*ZM0293* strain but not in ZM4 cells. The integrated expression of *ZMO0293* restored the growth of the glucose uptake-defective strain *Escherichia coli* BL21(DE3)-Δ*ptsG*. This study reveals the function of the *ZMO0293* gene in *Z. mobilis* in response to high concentrations of glucose and provides a new biological part for synthetic biology.

## 1. Introduction

Molasses and waste starch from potato factories is a promising feedstock for biofuel production [1]. Glucose is the major component liberated from these wastes during hydrolysis. The efficient utilization of glucose by microorganisms is crucial for economic biofuel production. *Zymomonas mobilis* is a Gram-negative α-proteobacterium with attractive physiological properties for bioethanol production. It can rely on the unique Entner Doudoroff (ED) pathway to anaerobically metabolize glucose to produce ethanol. Additionally, this ethanologenic bacterium has some desirable industrial characteristics, such as a high ethanol titer, high specific glucose uptake rate, high ethanol tolerance of 16% (*v*/*v*), and a broad pH range (3.5–7.5, especially low pH), which makes it an excellent chassis for the production of biochemicals [2,3,4,5]. Currently, different CRISPR–Cas systems have been established in *Z. mobilis* that facilitate gene editing manipulations [6,7,8,9]. Moreover, significant progress has been made to expand substrate ranges and enhance robustness against lignocellulosic biomass hydrolysate inhibitors through systems biology and metabolic engineering in *Z. mobilis*. In addition, various biological parts of synthetic biology, such as promoters, sRNAs, 5′ untranslated regions (5′ UTRs), and functional genes, have been characterized [10,11,12,13]. Metabolic models and assembly strategies have also been established to guide and construct metabolic pathways, which makes *Z. mobilis* a promising microbial biocatalyst for economic biosynthesis to produce value-added products such as lactate, isobutanol, and polyhydroxybutyrate (PHB) [14,15,16].

**Figure 1:**
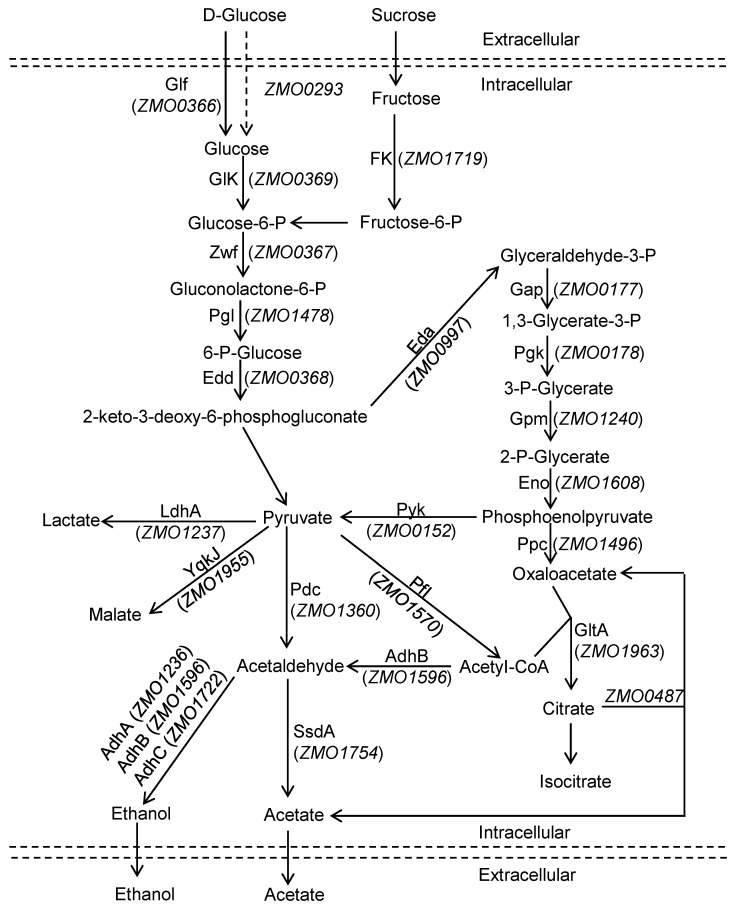
Sugar metabolism via the Entner Doudoroff (ED) pathway in *Z. mobilis. Z. mobilis* cells can utilize glucose, fructose, and sucrose via the ED pathway. Glucose is mainly transported by Glf (encoded by *ZMO0366*) and other sugar transporters, such as Zmo0293.

Molecular transport is a key process for cellular growth and metabolism. The transport of glucose and other sugars is the first step of glycolysis and is also an important rate-limiting step [17]. Different microorganisms have different types and characteristics of sugar transporters. In bacteria, glucose is transported through three main transport systems: phosphoenolpyruvate-dependent phosphotransferase systems (PTSs), the ATP-binding cassette (ABC) transport system, and the major facilitator superfamily (MFS), namely, H^+^/Na^+^ co- or unidirectional transporters [18]. In addition, *Saccharomyces cerevisiae* has a series of hexose transporter homologs, such as HxT1-17, Gal2, Snf3, and Rgt2, which have different substrate affinities and expression profiles [19,20]. Wild-type *Z. mobilis* only utilize glucose, sucrose, and fructose as carbon sources. Unlike most bacteria, *Z. mobilis* absorbs glucose by a unique facilitated diffusion mediated by the transmembrane transporter Glf (encoded by the *glf* gene, *ZMO0366*) (Figure 1) [21]. This facilitator is a low-affinity, high-velocity carrier that does not consume energy. Previous studies have shown that Glf could significantly increase the absorption rate of glucose, xylose, and fructose, alleviate the carbon catabolite repression (CCR) effect of the recombinant strain, and increase the yield of target products in *Escherichia coli* and *Corynebacterium glutamicum* [22,23]. Furthermore, metabolic engineering approaches have enabled xylose and arabinose metabolism in *Z. mobilis* [24,25]. Recently, transporter engineering strategies have played an increasingly important role in enhancing carbohydrate uptake, eliminating CCR, and improving hydrolysate inhibitor tolerance [26]. Although the main sugar transporter Glf has been identified in *Z. mobilis*, more sugar transporters still need to be characterized, and it is advantageous to understand their regulatory mechanism and develop efficient transporter engineering. *Z. mobilis* can rapidly ferment low concentrations of glucose (less than 100 g/L) and tolerates up to 400 g/L of glucose. Therefore, there is still room for improvement in ethanol fermentation at high concentrations by enhancing glucose transport capacity. Yang et al. found that the *ZMO0293* gene was downregulated in the presence of acetic acid or xylose and speculated that this protein might be a xylose transporter [27]. In addition, Zhang et al. also illustrated that the *ZMO0293* gene was significantly upregulated under conditions of adaptation to 220 g/L glucose [28]. However, the function of Zmo0293 has not been elucidated. It is necessary to understand the function of the sugar transporter Zmo0293 in central metabolism in *Z. mobilis*.

Here, we aimed to analyze the function of the sugar transporter gene *ZMO0293*. Firstly, we obtained the *ZMO0293* knockout mutant by the Type I-F CRISPR–Cas system and observed the influence on the growth rate, biomass, glucose consumption rate, and ethanol production rate. Secondly, qPCR was used to analyze the expression level of *ZMO0293* and other ED pathway genes. Finally, the *ZMO0293* gene was heterologously expressed to restore the growth of *E. coli* BL21(DE3)-Δ*ptsG*. Our study was helpful for identifying the function of the sugar transporter gene *ZMO0293* and provides a basis for understanding glucose uptake and sugar transporter engineering in *Z. mobilis*.

## 2. Results and Discussion

### 2.1. ZMO02393 Deletion Attenuated Growth and Fermentation Ability of ZM4 under High Glucose Concentrations

**Figure 2:**
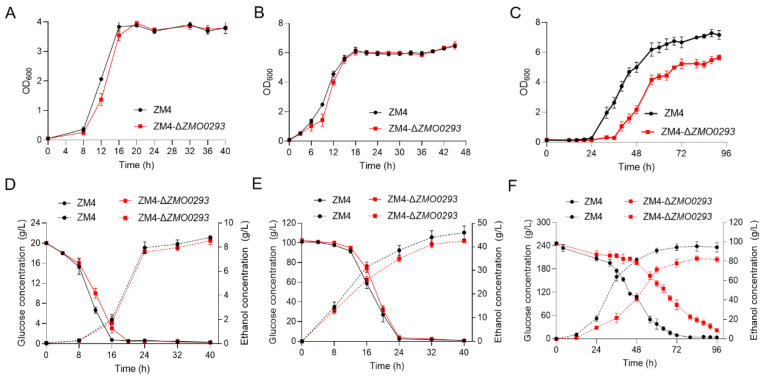
Effect of *ZMO0293* deletion on growth and ethanol fermentation. The fresh inoculums of ZM4 and ZM4-Δ*ZMO0293* were transferred to 50 mL RMG medium with 1% inoculum and cultivated at 30 °C. The effect of the *ZMO0293* gene on growth was evaluated by growth curve determination. (**A**–**C**) show the OD of ZM4 and ZM4-Δ*ZM0293* cultured in RMG medium supplemented with 20, 100, and 240 g/L glucose, respectively. The effect of the *ZMO0293* gene on ethanol fermentation was evaluated by glucose consumption and ethanol production. (**D**–**F**) show the glucose and ethanol concentrations of ZM4 and ZM4-Δ*ZMO0293* grown in RMG medium containing 20, 100, and 240 g/L glucose, respectively. The samples were taken at set time intervals. Data are presented as the mean ± s.d. with three biologically independent samples.

Sugar transporters are responsible for the import of sugars and play an important role in producing valuable biofuels and chemicals. Structure prediction of Zmo0293 using AlphaFold illustrated that this transporter possesses twelve transmembrane regions with high confidence, which is similar to that of Glf (Supplementary Figure S1). To reveal the function of Zmo0293, we deleted this gene in ZM4 cells by the endogenous Type I-F CRISPR–Cas system (Supplementary Figure S2) [6]. The final OD of the ZM4-Δ*ZMO0293* mutant reached 4.0 and 6.0 in the presence of 20 and 100 g/L glucose, which is similar to those of ZM4 (Figure 2A,B). However, this mutant showed an obvious growth defect in the presence of 240 g/L glucose. In particular, the final OD of ZM4-Δ*ZMO0293* was lower by 20.9% than that of the ZM4 control and had a longer lag phase of 12 h (Figure 2C).

When metabolizing the same amount of glucose or fructose, ATP production through the ED pathway in ZM4 is half of that through the Embden-Meyerhof-Parnas (EMP) pathway by other microorganisms, such as *S. cerevisiae*. Therefore, the ED pathway has less biomass accumulation, and most of the carbon sources (>95%) are converted to ethanol under low glucose concentrations [7,29]. We evaluated the role of the *ZMO0293* gene in ethanol fermentation. The ZM4-Δ*ZMO0293* mutant showed a slow glucose utilization rate and nearly the same ethanol titer in the presence of 20 g/L glucose compared to ZM4 (Figure 2D). The ZM4-Δ*ZMO0293* mutant exhibited a slow ethanol titer with almost the same glucose utilization rate in the presence of 100 g/L glucose (Figure 2E). However, when 240 g/L glucose was added, the glucose utilization rate and ethanol titer of the ZM4-Δ*ZMO0293* mutant were markedly slower than those of ZM4 (Figure 2F). After 96 h fermentation, the glucose consumption rate of ZM4-Δ*ZMO0293* was 2.3 g/L/h, 30.3% lower than that of ZM4 (3.3 g/L/h). Additionally, the ethanol rate of ZM4-Δ*ZMO0293* was 0.99 g/L/h, 34% lower than that of ZM4 (1.5 g/L/h).

*Z. mobilis* displays a long adaptation period under high concentrations of glucose or sucrose. Previous transcriptomic studies showed that ZM4 cells can regulate the transcription levels of genes associated with membrane channels and transporters, and metabolic pathways to adapt to high concentrations of glucose [28]. Our results showed that *ZMO0293* deletion (ZM4-Δ*ZMO0293*) can remarkably slow growth, glucose consumption rate, and ethanol production rate in the presence of a high glucose concentration (240 g/L), suggesting that the *ZMO0293* gene may function under high glucose concentrations.

**Figure 3:**
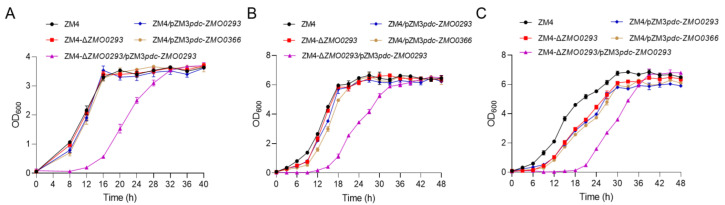
Effect of *ZMO0293* overexpression on growth. The growth curves of different strains were measured in RMG medium with 20 (**A**), 100 (**B**), and 200 (**C**) g/L glucose. The strain ZM4 represents WT, the strain ZM4-Δ*ZMO0293* for *ZMO0293* deletion, the strain ZM4/pZM3*pdc*-*ZMO0293* for the *ZMO0293* overexpression in ZM4, the strain *ZMO0366* ZM4/pZM3*pdc*-*ZMO0366* for the *ZMO0366* overexpression in ZM4, the strain ZM4-Δ*ZMO0293*/pZM3*pdc*-*ZMO0293* for the complementary strain of ZM4-Δ*ZMO0293* mutant.

Compared to the ZM4, the ethanol titer of ZM4-Δ*ZMO0293* cells was reduced by 8.3% and 7.1% under glucose concentrations of 100 and 240 g/L, respectively (Figure 2). These results suggested that *ZMO0293* deletion might conditionally affect glucose metabolism and ethanol production, especially under high glucose concentrations, which is consistent with previous results [28]. In addition, it is difficult to delete the *ZMO0366* (*glf*) gene by the CRISPR/Cas9 system used in this study and other methods, such as RecET [30], indicating that this gene is probably an essential gene for ZM4 cells. In order to test the potential of Zmo0293, we constructed the complementary strain by plasmid expression in ZM4-Δ*ZMO0293* (Supplementary Figure S3). When the glucose content in the RMG medium reached 240 g/L, the complementary strain of ZM4-Δ*ZMO0293* mutant (ZM4-Δ*ZMO0293*/pZM3*pdc*-*ZMO0293*) had a lag period of more than 30 h, which severely affected the normal growth. Therefore, the glucose concentration of 200 g/L was used to replace the 240 g/L. The results showed that the complementary strain of the ZM4-Δ*ZMO0293* mutant grew slowly and exhibited an obvious lag time (Figure 3C). Additionally, the OD of the *ZMO0293* and control *ZMO0366* overexpressed strain (ZM4/pZM3*pdc*-*ZMO0293* and ZM4/pZM3*pdc*-*ZMO0366*) was almost consistent with that of ZM4 and ZM4-Δ*ZMO0293* strains in the presence of 20 and 100 g/L glucose (Figure 3A,B). Under 200 g/L glucose, the growth of the overexpressed strain of the *ZMO0293* gene (ZM4/pZM3*pdc*-*ZMO0293*) was slower than that of ZM4 and the deletion strain (ZM4-Δ*ZMO0293*), and the final OD was 9.2 % and 7.8% lower than that of the ZM4 and ZM4-Δ*ZMO0293* strains, respectively (Figure 3C). It can be concluded that the overexpression effect of the *ZMO0293* gene did not work as expected in low or high glucose concentrations. A possible reason is that plasmid overexpression of sugar transporter Zmo0293 needs correct folding, localization, and insertion into the phospholipid bilayer. The copy number of plasmid pZM3 is higher than that of gene in the genome, which possibly leads to excessive protein expression and further affects the protein maturation process in the cell. In addition, the burden of plasmid expression on growth is another possible reason.

### 2.2. ZMO0293 Deletion Influences Transcriptional Changes in Some Genes of the ED Pathway

The ED pathway is responsible for glucose metabolism. Four genes, *glf* (*ZMO0366*), *zwf* (*ZMO0367*), *edd* (*ZMO0368*), and *glk* (*ZMO0369*), constitute an operon that enables efficient glucose utilization [31]. *glf* is the main sugar transporter for glucose uptake in ZM4. To examine the influence of *ZMO0293* deletion on the ED pathway, we determined the transcriptional level of the ED pathway by RT–qPCR. As shown in Table 1, after adding 240 g/L glucose, the relative expression level of *ZMO0293* in ZM4 increased from 1.05 (T1) to 1.49 (T2) and 2.58 (T3), while the expression level of *ZMO0366* decreased from 3.3 (T1) to −2 (T2) and −1.64 (T3). In the ZM4-Δ*ZM0293* strain, the expression level of *ZMO0366* decreased from 0.98 to −0.8 and −0.78. Although most ED pathway genes possess the same expression level, *ZMO1236* (*adhA*, encoding alcohol dehydrogenase), *ZMO1237* (*ldhA*, encoding D-lactate dehydrogenase), *ZMO1570* (*pflB*, encoding formate acetyltransferase), *ZMO0178* (*pgk*, encoding phosphoglycerate kinase), *ZMO1240* (*gpmA*, encoding 2,3-diphosphoglycerate-dependent phosphoglycerate mutase), and *ZMO1496* (*ppc*, encoding phosphoenolpyruvate carboxylase) displayed different expression levels in the ZM4-Δ*ZMO0293* strain in contrast to ZM4 cells.

The glucose uptake mediated by sugar transporters is usually the first step for glucose metabolism. ED pathway enzymes account for approximately 50% of total cellular proteins, together with pyruvate decarboxylase (PDC) and alcohol dehydrogenase B (ADHB), which facilitate rapid ethanol production in *Z. mobilis*. The influence of *ZMO0293* deletion on the ED pathway as measured by qPCR revealed that the inhibitory effect of *ZMO0366* on transcription levels by 240 g/L glucose addition was less than that of ZM4 (Table 1). In addition, glucose metabolism is usually involved in multiple functional genes and can provide energy and materials for other reactions. The expression level of multiple genes was influenced by deleting the *ZMO0293* gene, indicating that the sugar transporter Zmo0293 might be involved in central metabolism and the synthesis of the by-product lactic acid.

### 2.3. ZMO0293 Deletion Decreases the Activities of Key Enzymes Involved in Glucose Metabolism

**Figure 4:**
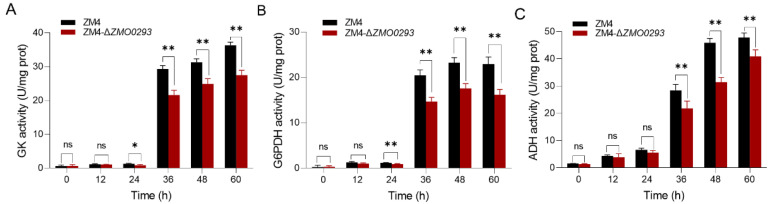
The enzymatic activities of GK, G6PDH, and ADH of ZM4 and ZM4-Δ*ZMO0293* in the presence of 240 g/L glucose. Enzymatic activities of GK (**A**), G6PDH (**B**), and ADH (**C**) were determined at the set time points. Data are presented as the mean ± s.d. from three independent experiments. * represents a statistically significant difference of *p* < 0.05 by Student’s *t* test; ** represents a statistically significant difference of *p* < 0.01; ns: no significant difference.

GK and G6PDH are the limiting enzymes of glucose utilization through the ED pathway, and ADH is the key enzyme of ethanol production. To characterize the effect of *ZMO0293* on the activities of glucose metabolism, three key enzymes (GK, G6PDH, and ADH) were selected, and their activities were measured in the presence of 240 g/L glucose. From Figure 4, the activities of these enzymes were similar in ZM4 and ZM4-Δ*ZMO0293* at 24-h fermentation. Nevertheless, these enzymatic activities of ZM4-Δ*ZMO0293* were gradually lower than those of ZM4. Maximal enzyme activities of GK, G6PDH, and ADH were reduced by 24.2% (60 h), 29.4% (60 h), and 31.4% (48 h), respectively.

It is noteworthy that almost all the enzymes of the ED pathway metabolize glucose near their maximal activities, and their activities are not regulated by feedback inhibition or other regulatory mechanisms [29]. The decreased enzymatic activities of GK, G6PDH, and ADH were also related to sugar transporter *ZMO0293* deletion (Figure 4). One explanation is that high osmotic stress resulting from high glucose concentrations is harmful to cell metabolism, and *ZMO0293* deletion is possibly detrimental to the absorption of glucose [32]. The molecular mechanism of the sugar transporter Zmo0293 and Glf in response to high glucose concentrations requires further elucidation in *Z. mobilis*.

### 2.4. Integration Expression of ZMO0293 can Promote the Growth of E. coli Strains Deficient in Glucose Uptake

**Figure 5:**
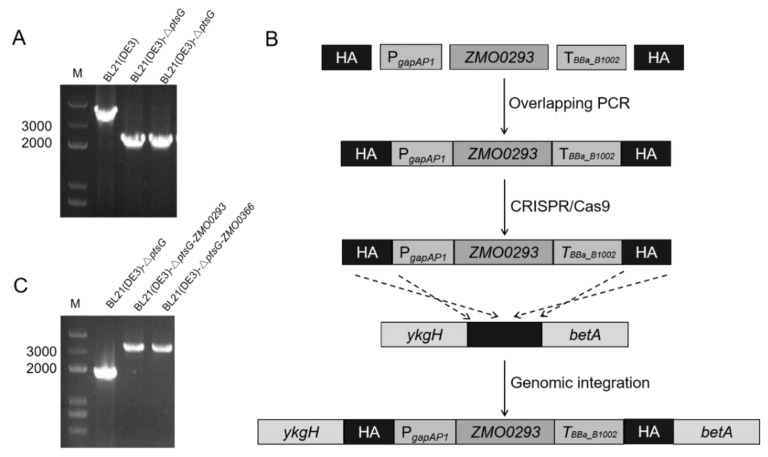
Integration expression of *ZMO0293* and *ZMO0366* in the *E. coli* BL21(DE3)-Δ*ptsG* strain. (**A**) The deletion of the *ptsG* gene was verified by PCR in the BL21(DE3) strain. (**B**), Schematic diagram of genomic integration of *ZMO0293* and *ZMO0366* expression cassettes using the CRISPR/Cas9 system. The gene expression cassette consisted of five parts: the weak promoter *gapAP1*, functional genes *ZMO0293* or *ZMO0366* and synthetic terminator *BBa_B1002*, the upstream and downstream homologous arms, taking the *ZMO0293* gene as an example. The gene expression cassette was inserted into the *ykgH*-*betA* neutral site in the genome of the *E. coli* BL21(DE3)-Δ*ptsG* strain. HA: homologous arm. (**C**) PCR verification of the integration of *ZMO0293* and *ZMO0366* expression cassettes.

**Figure 6:**
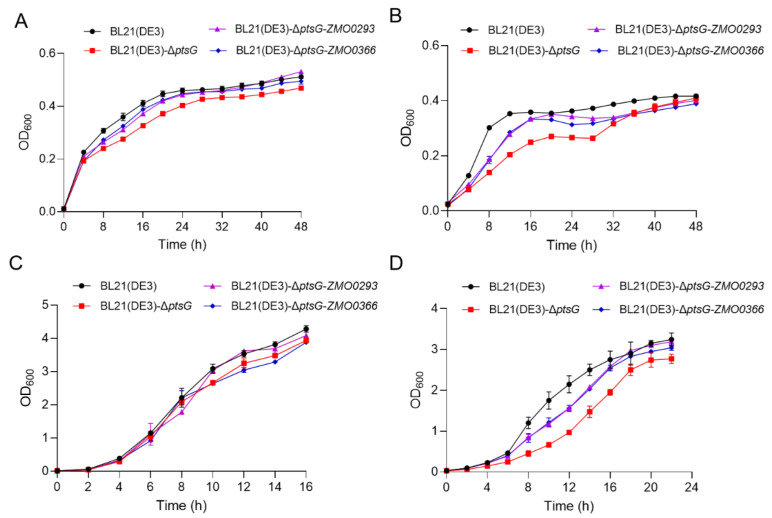
Effect of integration expression of *ZMO0293* and *ZMO0366* in the *E. coli* BL21(DE3)-Δ*ptsG* strain. BL21(DE3), BL21(DE3)-Δ*ptsG*, BL21(DE3)-Δ*ptsG*-*ZMO0293*, and BL21(DE3)-Δ*ptsG*-ZMO0366 were cultivated in 250 μL of LB (**A**) and 250 μL of M9 (**B**) media in Bioscreen machine, 30 mL of LB (**C**) and 30 mL of M9 (**D**) media in flasks with 1% inoculum. Error bars represent the s.d. of triplicate samples. BL21(DE3)-Δ*ptsG* indicated *ptsG* deletion in the *E. coli* BL21(DE3) strain, and BL21(DE3)-Δ*ptsG*-*ZMO0293* and BL21(DE3)-Δ*ptsG*-*ZMO0366* indicated *ZMO0293* and *ZMO0366* overexpression in the *E. coli* BL21(DE3)-Δ*ptsG* strain, respectively.

**Figure 7:**
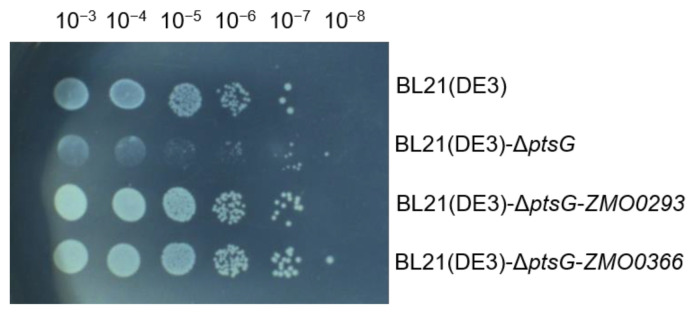
Spot assay of integration expression of *ZMO0293* and *ZMO0366* in *E. coli* BL21(DE3) strains. The initial OD_600_ of all strains was adjusted to 1.0, and these *E. coli* cells were serially diluted tenfold. All the strains were spotted on M9 agar plates and cultivated at 37 °C.

Sugar transporters are useful functional genes in accelerating sugar absorption, reducing intracellular PEP (phosphoenolpyruvate) consumption, and alleviating CCR [23,33]. *E. coli* uses the PTS to transport extracellular glucose into the cells [34]. To explore the application potential of the sugar transporter Zmo0293, we deleted the BL21(DE3) *ptsG* gene using CRISPR/Cas9 to obtain the BL21(DE3)-Δ*ptsG* strain, which is deficient in glucose absorption (Figure 5A). Chromosome integration of functional genes is more stable than plasmid expression [35]. Therefore, *ZMO0293* and *ZMO0366* genes under the weak *E. coli gapAP1* promoter were integrated into the *ykgH*-*betA* neutral site of the genome of BL21(DE3)-Δ*ptsG*, respectively (Figure 5B,C). The medium type and cultivation volume were important factors affecting the function of gene expression. Here, we employed LB and M9 medium, together with flasks and 96-well plates, to evaluate the role of Zmo0293 and Glf in *E. coli* BL21(DE3)-Δ*ptsG*. The results showed that the growth of the BL21(DE3)-Δ*ptsG* strain was slower than that of the control BL21(DE3) strain in LB or M9 medium (Figure 6). Furthermore, the integration expression of *ZMO0293* and *ZMO0366* can almost restore the growth of the BL21(DE3) *ptsG* deletion strain. We also found that the restoration of Zmo0293 is similar to that of Glf. In addition, the recovery performance of these two genes in the M9 medium was better than that in the LB medium. Subsequently, the effect of the integration expression of *ZMO0293* in the BL21(DE3)-Δ*ptsG* strain was further evaluated by a spot assay. The results also showed that the *ZMO0293* gene can improve the cell growth of BL21(DE3)-Δ*ptsG* in the M9 agar medium (Figure 7).

It has been demonstrated that glucose transport in recombinant *E. coli* is facilitated by the glucose transporter Glf from *Z. mobilis* [36]. The growth of BL21(DE3)-Δ*ptsG* was promoted by the integrated expression of *ZMO0293* driven by the weak promoter *gapAP1*, which is comparable to that of *ZMO0366* (*glf*). Medium type (LB and M9) can possibly affect the function of Zmo0293 and Glf in *E. coli* BL2(DE3)-Δ*ptsG*, for which one explanation is that LB medium contains more rich nutrition than M9 (Figure 5 and Figure 6). Enhanced growth ability will be beneficial for glucose utilization in *E. coli* BL2(DE3)-Δ*ptsG*.

Mining sugar transporters can provide useful targets for genetic engineering and synthetic biology. For example, other studies proved that characterizing and engineering sugar uptake system can enhance the titer of milbemycins in *Streptomyces bingchenggensis* [37]. The expression of a xylose transporter xylE from *E. coli* could enhance the rate of xylose utilization in *Z. mobilis* at high xylose concentrations [38]. Nevertheless, the lack of a dedicated xylose transport system in recombinant *Z. mobilis* thus limits the capacity for dual xylose and glucose fermentation as well as the high xylose catabolic pathway. This bottleneck highlights the necessity to identify and engineer efficient xylose transport proteins in *Z. mobilis*. The absorbable carbohydrate spectrum and absorption kinetics of sugar transporter Zmo0293 need to be further studied.

## 3. Materials and Methods

### 3.1. Strains, Media, and Cultivation Conditions

*Zymomonas mobilis* ZM4 (ATCC31821) and its derivatives were cultured in a rich medium (RMG, 2% glucose, 1% yeast extract, 0.2% KH_2_PO_4_, and pH 5.8) at 30 °C. The antibiotic spectinomycin (100 μg/mL) was added when required [6,28].

*E. coli* BL21(DE3) and its derivatives were cultured in Luria–Bertani medium (LB, 1% tryptone, 0.5% yeast extract, 1% NaCl, and pH 7.0) or M9 medium (2% glucose, 0.6% Na_2_HPO_4_, 0.3% KH_2_ PO_4_, 0.05% NaCl, 0.1% NH_4_Cl, 1 mM MgSO_4_, 0.1 mM CaCl_2_, and 0.1% (*v*/*v*) 1000 × Mixed solution of the following trace elements: 2.7% FeCl_3_·6H_2_O, 0.2% ZnCl_2_·4H_2_O, 0.2% CaCl_2_·2H_2_O, 0.2% Na_2_MoO_4_·2H_2_O, 1.9% CuSO_4_·5H_2_O, and 0.5% H_3_BO_3_) at 37 °C. Antibiotics were used at the following final concentrations: spectinomycin (100 μg/mL) and kanamycin (50 μg/mL). *E. coli* DH5α was used for plasmid construction, and BL21(DE3) was used for mutant and recombinant construction.

### 3.2. Construction of ZMO0293 Mutant Using a Native CRISPR-Cas System in ZM4

#### 3.2.1. Plasmid Construction

The *ZMO0293* gene was deleted by a native CRISPR–Cas genome editing system [6]. Briefly, the editing plasmids were initially constructed with a spacer containing a 5′-CCC-3′ PAM. The editing plasmid backbone was amplified by PCR. Oligonucleotides were annealed by first heating the reaction mixture to 95 °C for 5 min and subsequently cooling gradually to room temperature. The annealed spacer, the upstream and downstream homologous arms (HAs) of *ZMO0293* with a length of 500–1000 bp, and the plasmid backbone were linked by seamless cloning (Vazyme, Nanjing, China), and then the reaction product was introduced into *E. coli* DH5α competent cells to generate the pMini-Δ*ZMO0293* editing plasmid. Correct plasmids were verified by colony PCR and Sanger sequencing. pZM3*pdc-ZMO0366* and pZM3*pdc-ZMO0366* were also constructed by seamless cloning using pZM3*pdc* as plasmid backbone [30]. All strains, plasmids, and primers used in this study are listed in Table 2 and Supplementary Tables S1 and S2.

#### 3.2.2. Electroporation and Mutant Selection

The pMini-Δ*ZMO0293* plasmid was transformed into ZM4 competent cells (1 μg DNA with 50 μL competent cells) via electroporation using a Bio-Rad Gene Pulser (Bio-Rad, Hercules, CA, USA). The electroporation parameters were as follows: 0.2 cm electroporation cuvettes, 1.6 kV, 25 μF, and 200 Ω. The electroporated cells were immediately transferred to 1 mL RMG and recovered at 30 °C for 8–12 h. The cells were then spread on RMG agar plates containing appropriate antibiotics and incubated at 30 °C for 2–3 days to isolate single colonies. Colonies with correct plasmids were cultivated in an RMG medium containing appropriate antibiotics and verified by colony PCR.

### 3.3. Construction of Recombinant Strains in E. coli Using the CRISPR/Cas9 System

*E. coli* BL21(DE3) was used as the host for genomic manipulations. Gene deletion and integration expression were performed by the CRISPR/Cas9 system with two plasmids, one plasmid, pTargetF (Addgene number 62226), was responsible for target site recognition, and the other plasmid, pEcCas (Addgene number 73227), was used for target site cutting [39,40]. When needed, both spectinomycin and kanamycin were used at final concentrations of 50 μg/mL. L-arabinose and rhamnose were added at concentrations of 10 mM and 10 mM, respectively.

#### 3.3.1. Plasmid Construction

The pTargetF plasmid used for deletion and integration of the target site was constructed by a pair of primers with the gRNA spacer sequence specific for each target and the flanked sequences homologous to the pTargetF backbone (Table 2, Supplementary Table S1). The resultant PCR products were transformed into DH5α competent cells after *Dpn* I digestion and then ligated via homologous recombination to form pTargetF-*ptsG* and pTargetF-*ykgH*-*betA*.

#### 3.3.2. Donor Construction

The upstream and downstream HAs with a length of 500–1000 bp were fused by overlap extension PCR to form a donor DNA for seamless deletion of the *ptsG* gene. The expression donor for *ZMO0293* and *ZMO0366* was constructed for chromosome integration [41]. These two genes, *ZMO0293* and *ZMO0366*, were driven by the promoters P*_gapAP1_* and synthetic terminator T*_BBa_B1002_*, respectively. The HA length at both ends was also 500–1000 bp.

#### 3.3.3. Electroporation and Screening of Mutants

Briefly, 40 μL electrocompetent cells harboring the pEcCas plasmid, 400 ng donor DNA, and 100 ng gRNA plasmid were mixed for electroporation in a Gene Pulser^®^ II electroporator (Bio-Rad Laboratories, Hercules, CA, USA) using the following parameters: 1.0 mm cuvette gap, 1.8 kV, 200 Ω, and 25 μF [40]. After electroporation, the transformed cells were immediately resuspended in 1 mL of LB and allowed to recover at 37 °C for 1 h prior to plating on LB agar supplemented with kanamycin and spectinomycin. After overnight culture at 37 °C, the single colonies were randomly picked for colony PCR. The selected transformants were further confirmed by Sanger sequencing. The plasmids pEcCas9 and pTargetF could be cured when the strain was not subjected to further engineering.

### 3.4. Growth Curve Determination

Three single colonies of *Z. mobilis* were picked and cultivated in 5 mL RMG medium overnight at 30 °C without agitation. The cultures were transferred to 30 mL RMG medium at 1% inoculum. Three single colonies of *E. coli* were picked and cultivated in 5 mL of LB or M9 medium overnight at 37 °C and 220 rpm. The cultures were transferred to 30 mL LB or M9 medium in flasks or 250 μL LB or M9 medium in a 96-well plate for cultivation in a BioScreen Automated Microbial Growth Analyzer (Oy Growth Curves Ab Ltd., Helsinki, Finland) with 1% inoculum. Cell growth was monitored by measuring the absorbance at 600 nm (OD_600_).

### 3.5. Real-time Quantitative PCR

ZM4 and ZM4-Δ*ZMO0293* strains were initially grown in RMG medium in the presence of 20 g/L glucose. When the OD_600_ values reached 0.4, the samples were removed (T1) and immediately frozen in liquid nitrogen. Simultaneously, glucose was added to a final concentration of 240 g/L. After 20 min and 40 min of treatment, the samples served as T2 and T3, respectively. The samples without glucose addition were chosen as the control. The frozen samples were extracted to obtain RNA according to the manufacturer’s protocol (Tiangen, Beijing, China).

Real-time quantitative PCR (qPCR) analysis was performed to evaluate ED pathway gene expression levels. The RNA samples were reverse-transcribed by PrimeScript^®^ RT Enzyme Mix I (Takara Biotechnology-Dalian Co., Ltd., Dalian, China). The sequences of the primer pairs are listed in Supplementary Table S3. The gene *rrsA* (*ZMOr009*), encoding the 16S ribosomal RNA, was used as a control gene. qPCR was performed using an iQ5 Real-time PCR System (Bio-Rad Laboratories, Inc., Hercules, CA, USA) with 2×TaKaRa SYBR Green Real-Time PCR Master Mix (Takara Biotechnology-Dalian Co., Ltd., Dalian, China). The data were calculated using the 2^−ΔΔCT^ method [42].

### 3.6. Fermentation Analysis

RMG medium (50 mL) was added to a 250 mL Erlenmeyer flask. The final glucose concentration was adjusted to reach 20 g/L, 100 g/L, and 240 g/L. Fermentation was carried out without agitation at 30 °C with inoculation of 1% preculture. During the culture process, samples were taken every 4 h to determine the OD value. The glucose concentration in the fermentation broth was determined by the 3,5-dinitrosalicylic acid method [43]. The ethanol content was analyzed using an ethanol determination kit according to the manufacturer’s instructions (Suzhou Grace Biotechnology Co., Ltd., Suzhou, China).

### 3.7. Determination of Enzyme Activities

ZM4 and ZM4-Δ*ZMO0293* were cultivated at 30 °C in an RMG medium containing 240 g/L glucose. The culture was centrifuged for 15 min at 12,000 rpm. The pellets were washed with a sterile physiological saline solution. The samples were pretreated according to the method by Algar and Scopes [29]. The activities of the enzymes glucokinase (GK), glucose-6-phosphate dehydrogenase (G6PDH), and alcohol dehydrogenase (ADH) were measured according to the manufacturer’s instructions (Suzhou Grace Biotechnology Co., Ltd., Suzhou, China).

### 3.8. Spot Assay of Various E. coli BL21(DE3) Strains

*E. coli* BL21(DE3) and its derivatives were cultured in an M9 medium at 37 °C for 24 h. The OD_600_ of various cultures was adjusted to the same level, and 2.0 µL of the tenfold diluted suspensions of each strain was spotted on solid M9 medium. The plates were incubated at 37 °C for 2 days.

## 4. Conclusions

The deletion of the sugar transporter-encoding gene *ZMO0293* can result in growth defect, low glucose consumption rate, and ethanol titer in *Z. mobilis*. It can also cause the low activities of key enzymes (GK, G6PDH, and ADH) for glucose metabolism and differential expression of some genes of the ED pathway. In addition, it can restore the growth of *E. coli* BL21(DE3)-Δ*ptsG* deficient in glucose absorption. The results of this study are not only helpful for metabolic engineering regulation of glucose utilization but are also beneficial to alleviate or eliminate the CCR phenotype of recombinant strains in the future.

## Figures and Tables

**Table 1:** The relative expression levels of ED pathway genes.

Locus Tag	Gene	Function	Fold Change *					
			ZM4			ZM4-Δ*ZM0293*		
			T1	T2	T3	T1	T2	T3
*ZMO0293*	*—*	sugar transporter	1.05	1.49	2.58	*—*	*—*	*—*
*ZMO0366*	*glf*	glucose facilitator	3.3	−2	−1.64	0.98	−0.8	−0.78
*ZMO0367*	*zwf*	glucose-6-phosphate dehydrogenase	0.98	−0.04	0.11	1	0.13	0.63
*ZMO0368*	*edd*	phosphogluconate dehydratase	1.02	1.85	0.27	1	3.29	5.13
*ZMO0369*	*glk*	glucokinase	1	0.22	0.09	1.01	0.45	1.68
*ZMO1236*	*adhA*	alcohol dehydrogenase	1	−0.96	−0.59	1	−0.23	1.32
*ZMO1237*	*ldhA*	D-Lactate dehydrogenase	1.06	−0.9	0.51	1	0.97	6.14
*ZM01754*	*ssdA*	aldehyde dehydrogenase (NAD^+^)	1.05	1.14	0.73	1	1.53	4.85
*ZMO1722*	*adhC*	class III alcohol dehydrogenase	0.91	−0.39	−0.50	1	−0.63	−0.33
*ZMO1719*	*frk*	fructokinase	1.08	−0.8	−0.76	1.06	−0.68	−0.33
*ZMO0997*	*eda*	2-dehydro-3-deoxy-phosphogluconate aldolase	1.01	−0.49	−0.18	1	−0.49	−0.02
*ZMO0487*	*—*	CoA ester lyase	1	−0.54	−0.51	1.01	−0.71	−0.72
*ZMO1570*	*pflB*	formate acetyltransferase	0.96	−0.27	−0.26	1	−0.36	0.12
*ZMO1955*	*yqkJ*	NAD-dependent malic enzyme	1	−0.86	−0.83	1	−0.61	−0.28
*ZMO1240*	*gpmA*	2,3-diphosphoglycerate-dependent phosphoglycerate mutase	0.96	−0.66	−0.61	1.01	−0.32	0.3
*ZMO1478*	*pgl*	6-phosphogluconolactonase	1.04	−0.59	−0.62	1	−0.61	−0.48
*ZMO0178*	*pgk*	phosphoglycerate kinase	1.07	−0.88	−0.88	1	1.01	1.80
*ZM01963*	*gltA*	citrate synthase	1.01	−0.67	−0.59	1	−0.35	−0.48
*ZMO0177*	*gap*	glyceraldehyde-3-phosphate dehydrogenase, type I	1	0.33	0.18	1	0.05	0.68
*ZMO1608*	*eno*	phosphopyruvate hydratase	1	−0.47	−0.57	1	−0.62	−0.21
*ZMO0152*	*pyk*	pyruvate kinase	1	−0.53	−0.48	1	−0.59	−0.28
*ZMO1496*	*ppc*	phosphoenolpyruvate carboxylase	1	−0.14	−0.01	1	0.31	0.73
*ZMO1596*	*yiaY*	L-threonine dehydrogenase	0.98	0.09	0.19	1	0.2	1.11
*ZMO1360*	*pdc*	alpha-keto acid decarboxylase family protein	0.97	−0.80	−0.77	1	−0.75	−0.65
*ZMO0569*	*sdhC*	succinate dehydrogenase cytochrome b subunit	1.02	0.03	−0.14	1	−0.56	−0.31

*: The data from qPCR were transformed with log2. T1, T2, and T3 represent ZM4 and ZM4-Δ*ZMO0293* cells treated with 240 g/L glucose for 0, 20, and 40 min, respectively. Data were from three replicates.

**Table 2:** Strains and plasmids used in this study.

Name	Description	Sources
Strains		
ZM4	Wild type	Lab stock
ZM4-Δ*ZMO0293*	ZM4, Δ*ZMO0293*	This study
ZM4/pZM3*pdc-ZMO0293*	ZM4, P*pdc*-*ZMO0293*	This study
ZM4/pZM3*pdc-ZMO0366*	ZM4, P*pdc*-*ZMO0366*	This study
ZM4-Δ*ZMO0293*/pZM3*pdc-ZMO0293*	ZM4, Δ*ZMO0293*, P*pdc*-*ZMO0293*	This study
DH5α	/	Lab stock
BL21(DE3)	/	Lab stock
BL21(DE3)-Δ*ptsG*	BL21(DE3), Δ*ptsG*	This study
BL21(DE3)-Δ*ptsG*-*ZMO0293*	BL21(DE3), Δ*ptsG*, *ykgH*-*betA::*P*_gapAP1_*- *ZMO0293*-T*_BBa_B1002_*	This study
BL21(DE3)-Δ*ptsG*-*ZMO0366*	BL21(DE3), Δ*ptsG*, *ykgH*-*betA::*P*_gapAP1_*- *ZMO0366* -T*_BBa_B1002_*	This study
Plasmids		
pTargetF	P*_J23119_*-N20-sgRNA scaffold, *Sm^R^*, *pMB1 ori*	[39]
pEcCas9	P*_cas_*-*Cas9*, *araC*-P*_araB_*-Red, *rhaRS*-P*_rhaB_*- sgRNA(pMB1), *pSC101 ori*, *sacB*, *kana^R^*	[40]
pTarget-Δ*ptsG*	Derived from pTargetF, targeting *ptsG* in *E. coli* BL21(DE3)	This study
pTarget-*ykgH*-*betA*	Derived from pTargetF, targeting *ykgH*-*betA* neutral site in *E. coli* BL21(DE3)	This study
pMini	P*_Psp_-cat*, *P15A ori*, *Zymo ori*, *SpCas9*, miniCRISPR	[6]
pMini-Δ*ZMO0293*	Derived from pMini, targeting *ZMO0293* in ZM4	This study
pZM3*pdc-ZMO0366*	*Zymo P2ori*, *kana^R^*, P*pdc* from ZM4, used for overexpression of *ZMO0366*	This study
pZM3*pdc-ZMO0293*	*Zymo P2ori*, *kana^R^*, P*pdc* from ZM4, used for overexpression of *ZMO0293*	This study

## Data Availability

The data presented in this study are available on request from the corresponding author.

## Supplementary Materials

The following supporting information can be downloaded at: https://www.mdpi.com/article/10.3390/ijms24065888/s1.
