# Supplementary material for: Characterization and Application of the Sugar Transporter Zmo0293 from Zymomonas mobilis
Source: Int J Mol Sci. 2023 Mar 20;24(6):5888. doi: 10.3390/ijms24065888 (PMC10055971; doi:10.3390/ijms24065888)
Supplement: Supplementary file 1 [file ijms-24-05888-s001.zip › ijms-2228786-supplementary.pdf]

Supplementary information for

Characterization and application of the sugar transporter Zmo0293 from *Zymomonas mobilis*

Kun Zhang, Wenwen Zhang, Mengxing Qin, Yi Li, Hailei Wang*

Henan Province Engineering Laboratory for Bioconversion Technology of Functional Microbes, College of Life Sciences, Henan Normal University, Xinxiang 453007, Henan, China

* Correspondence: whl@htu.cn (H. Wang)

Supplementary Figure S1. Comparison of structure prediction of Zmo0293 and Glf. Structure prediction was performed using AlphaFold. A. The predicted transmembrane region of Zmo0293; B. The predicted 3D structure of Zmo0293; C. The predicted transmembrane region of Glf; D. The predicted 3D structure of Glf.

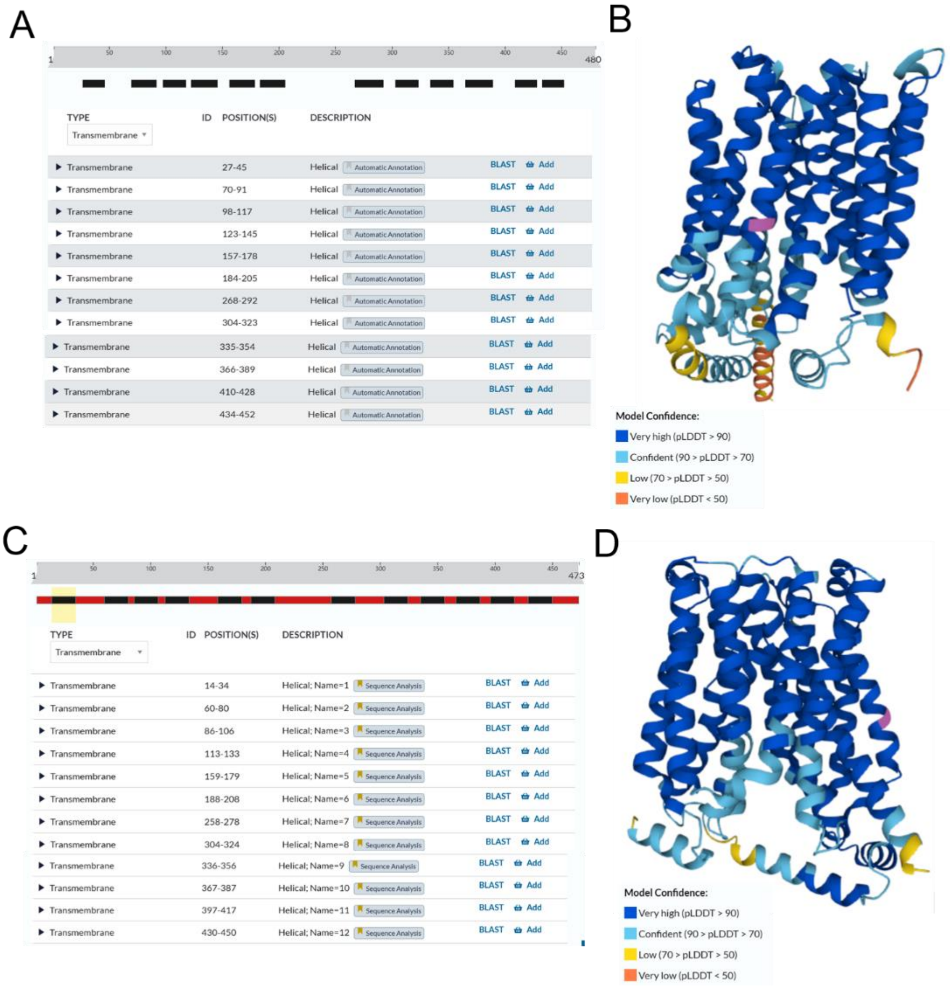

AlphaFold website: https://www.alphafold.ebi.ac.uk/

Supplementary Figure S2. PCR verification of deletion of *ZMO0293* gene in ZM4.

Lanes 1 and 2 represent wild -type ZM4; Lanes 3 and 4 represent ZM4-Δ*ZMO0293* mutant;

Lanes 5 and 6 represent *ZMO0293* fragment in ZM4-Δ*ZMO0293* mutant.

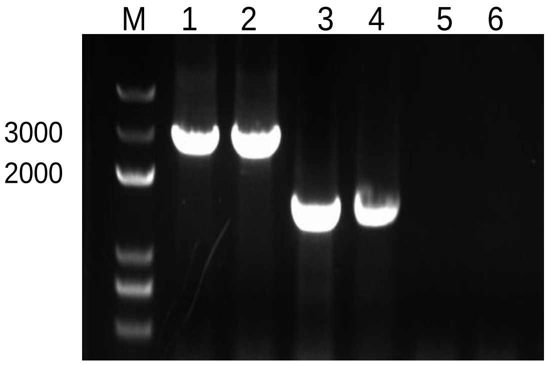

Supplementary Figure S3. PCR verification of overexpression of *ZMO0293* gene in ZM4. Lane 1 represents wild -type ZM4; Lane 2 represents ZM4-Δ*ZMO0293*/pZM3*pdc*- *ZMO0293*. Complement strain was constructed in ZM4-Δ*ZMO0293* with plasmid expression of *ZMO0293*.

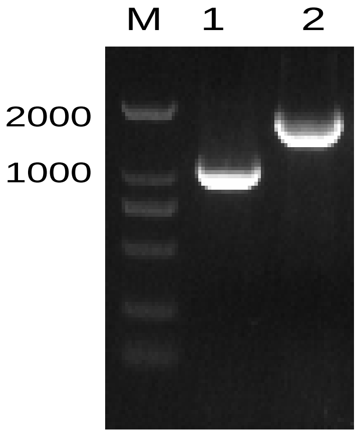

Supplementary Table S1. Primer used in this study

**Primers**	**Sequences (5'- 3')**
**Genome modification for knockout of *ptsG* gene in *E. coli*:**	
ptsGsg-F	CGACATTCCGCGTTATATGGGTTTTAGAGCTAGAAATAGC
ptsGsg-R	CCATATAACGCGGAATGTCGACTAGTATTATACCTAGGAC
ptsGUP-F	GGTAGGCGAACGTGACAACG
ptsGUP-R	AATTGAGAGTGCTCCTGAGTATGG
ptsGDN-F	AAGCACCCATACTCAGGAGCACTCTCAATTTCCGTAAGACGTT GGGGAGAC
ptsGDN-R	CGCAGGACGATACCACACATATG
ptsG-TF	AGTCGACTCACACTGCCATCTC
ptsG-TR	CGGTTGGAACAGTGGCAAACG
**Genome modification for integrating the *ZMO0293* gene in the neutral site:**	
ycgH-betA-sg-F	CATCATGAATACTGGATATGGTTTTAGAGCTAGAAATAGC
ycgH-betA-sg-R	CATATCCAGTATTCATGATGACTAGTATTATACCTAGGAC
ykgH-UP-F	AATCCCAGAGCCAGGCAAAT
ykgH-UP-R	ACCCGAACATTCCTGATACAG
betA-DN-F	CCTGCGTTAGTTCACATCACG
betA-DN-R	ACGGCCAACAAAGAAGTGCT
gapAP1-F	ATAGTCTGTATCAGGAATGTTCGGGTGAGGCGAGTCAGTCGC GTAATG
gapAP1-R	CCTCCTTTCCACCAGCTATTTGTTAGTGAATAAAAG
ZMOO293-F1	AATAACAATTCAGAAATAGAAAATGATACCC
ZMOO293-R1	GCGAAAAAACCCCGCCGAAGCGGGGTTTTTTGCGTTAGGCGG AGGCTTTTTTACTC
ZMOO293-F2	CACTAACAAATAGCTGGTGGAAAGGAGGAATAACAATTCAGA AATAGAAAATGATACCC
ZMOO293-R2	AAATGAGTCGTGATGTGAACTAACGCAGGGCGAAAAAACCCC GCCGAAG

Genome modification by knockout of *ZMO0293* gene in ZM4:

ZMO0293sg-F	GGCATTGAAATGATGATCTATTATACCCCGACGTTCACTGCCG CACAGGCAGCTTAGAAAGGATCCTCGAACGCGCCGAAT
ZMO0293sg-R	ATTCGGCGCGTTCGAGGATCCTTTCTAAGCTGCCTGTGCGGCA GTGAACGTCGGGGTATAATAGATCATCATTTCAATGCC
mini-F	GGATCCTCGAACGCGCCGAATAAGTAATTCAGGTTTTTTATA
mini-R	AATAGATCATCATTTCAATGCCTTTCTAAGCTGCCTGTGCGGC AGTGAACGGTACCAGA
ZMO0293UP-F	TACCGGCTATATCAAGCTGCAAGTGTCGACCGAATAGCCCCTG CATAGCG
ZMO0293UP-R	ACCGGATTTTATAAAAAATAGATTATGCTGAACTAGAAATTTAT CCCTTTTTTTAGCG
ZMO0293DN-F	TTCAGCATAATCTATTTTTTATAAAATCCG
ZMO0293DN-R	CAGTGTGACCTGCAGCGGCCGCTACTAGTAATCATCCGTTTGT TCCGGTT
pMini-F	TACTAGTAGCGGCCGCTGCAG
pMini-R	GTCGACACTTGCAGCTTGAT
ZMO0293-DF	TAAAGACCTGTTGCGGGAGG
ZMO0293-DR	TAGCGAGTCAGTGAGCGAGG
ZMO0293-TF	CCGCCCAAGGCATTGTACTG
ZMO0293-TR	AGCTTTCTACACGAGCTCTT
pdcZMO0293-F	ACATAGTGTTTTGAATATATGGAGTAAGCAATGAATAACAATTC AGAAATAGAAAATGATACC
pdcZMO0293-R	ATCGCCATGTAAGCCCACTGCAAGCTACCTTTAGGCGGAGGC TTTTTTACTC
pdcZMO0366-F	ACATAGTGTTTTGAATATATGGAGTAAGCAATGAGTTCTGAAA GTAGTCAGGGTCTAGTC
pdcZMO0366-R	ATCGCCATGTAAGCCCACTGCAAGCTACCTCTACTTCTGGGAG CGCCACATCTCC

pZM3pdc-F	AGGTAGCTTGCAGTGGGCTT
pZM3pdc-R	TGCTTACTCCATATATTCAAAACACTATGTCTG
pZM3pdc-TF	GTTACGCTCATGATCGCGGC
pZM3pdc-TR	ATCAGATCTTGATCCCCTGCG

Supplementary Table S2. The plasmid sequence used in this study.

Name	sequence
pMini	CGTTCACTGCCGCACAGGCAGCTTAGAAAGGCATTGAAATGATGATCTATTATACCCCGAC<br>GTTCACTGCCGCACAGGCAGCTTAGAAAGGATCCTCGAACGCGCCGAATAAGTAATTCAG<br>GTTTTTTTATAAAGACCTGTTGCGGGAGGTGGCCATGGGGGTCATCGTCAACCGCTTAACC<br>TGAAAAGGGTGATAAAATGGCAAAAAAAATTACCGGCTATATCAAGCTGCAAGTGTCGAC<br>TACTAGTAGCGGCCGCTGCAGGTCACACTGGCTCACCTTCGGGTGGGCCTTTCTGCGTTTA<br>TATACTAGAGAGAGAATATAAAAAGCCAGATTATTAATCCGGCTTTTTTATTATTTGCGCTA<br>GCGGAGTGTATACTGGCTTACTATGTTGGCACTGATGAGGGTGTCAGTGAAGTGCTTCATG<br>TGGCAGGAGAAAAAAGGCTGCACCGGTGCGTCAGCAGAATATGTGATACAGGATATATTC<br>CGCTTCCTCGCTCACTGACTCGCTACGCTCGGTCGTTCGACTGCGGCGAGCGGAAATGGC<br>TTACGAACGGGGCGGAGATTTCCTGGAAGATGCCAGGAAGATACTTAACAGGGAAGTGA<br>GAGGGCCGCGGCAAAGCCGTTTTTCCATAGGCTCCGCCCCCCTGACAAGCATCACGAAAT<br>CTGACGCTCAAATCAGTGGTGGCGAAACCCGACAGGACTATAAAGATACCAGGCGTTTCC<br>CCCTGGCGGCTCCCTCGTGCGCTCTCCTGTTCCTGCCTTTCGGTTTACCGGTGTCATTCCG<br>CTGTTATGGCCGCGTTTGTCTCATTCCACGCCTGACACTCAGTTCCGGGTAGGCAGTTCGC<br>TCCAAGCTGGACTGTATGCACGAACCCCCCGTTCAGTCCGACCGCTGCGCCTTATCCGGT<br>AACTATCGTCTTGAGTCCAACCCGGAAAGACATGCAAAAGCACCACTGGCAGCAGCCAC<br>TGGTAATTGATTTAGAGGAGTTAGTCTTGAAGTCATGCGCCGGTTAAGGCTAAACTGAAA<br>GGACAAGTTTTGGTGACTGCGCTCCTCCAAGCCAGTTACCTCGGTTCAAAGAGTTGGTAG<br>CTCAGAGAACCTTCGAAAAACCGCCCTGCAAGGCGGTTTTTTCGTTTTCAGAGCAAGAGA<br>TTACGCGCAGACCAAAACGATCTCAAGAAGATCATCTTATTAATCAGATAAAATATTTCTTG<br>ATTTCAGTGCAATTTATCTCTTCAAATGTAGCACCTGAAGTCAGCCCCATACGATATAAGTT<br>GTAAATTCTCATGTTTGACAGCTTATCATCGATGGAGCACAGGATGACGCCTAACAATTCA<br>TTCAAGCCGACACCGCTTCGCGGCGCGGCTTAATTCAGGAGTTAAACATCATGAGGGAAG<br>CGGTGATCGCCGAAGTATCGACTCAACTATCAGAGGTAGTTGGCGTCATCGAGCGCCATCT<br>CGAACCGACGTTGCTGGCCGTACATTTGTACGGCTCCGCAGTGGATGGCGGCCTGAAGCC<br>ACACAGTGATATTGATTTGCTGGTTACGGTGACTGTAAGGCTTGATGAAACAACGCGGCG<br>AGCTTTGATCAACGACCTTTTGGAAACTTCGGCTTCCCCTGGAGAGAGCGAGATTCTCCG<br>CGCTGTAGAAGTCACCATTGTTGTGCACGACGACATCATTCCGTGGCGTTATCCAGCTAAG<br>CGCGAACTGCAATTTGGAGAATGGCAGCGCAATGACATTCTTGCAGGTATCTTCGAGCCA<br>GCCACGATCGACATTGATCTGGCTATCTTGCTGACAAAAGCAAGAGAACATAGCGTTGCC<br>TTGGTAGGTCCAGCGGCGGAGGAACTCTTTGATCCGGTTCCTGAACAGGATCTATTTGAG<br>GCGCTAAATGAAACCTTAACGCTATGGAACTCGCCGCCCGACTGGGCTGGCGATGAGCGA<br>AATGTAGTGCTTACGTTGTCCCGCATTTGGTACAGCGCAGTAACCGGCAAAATCGCGCCG<br>AAGGATGTCGCTGCCGACTGGGCAATGGAGCGCCTGCCGGCCCAGTATCAGCCCGTCATA<br>CTTGAAGCTAGGCAGGCTTATCTTGGACAAGAAGATCGCTTGGCCTCGCGCGCAGATCAG<br>TTGGAAGAATTTGTTCACTACGTGAAAGGCGAGATCACCAAGGTAGTCGGCAAATAATGT<br>CTAACAATTCGTTCAAGCCGACGCCGCTTCGCGGCGCGGCTTAACTCAAGCGTTAGAGAG<br>CTGGGGAAGACTATGCGCGATCTGTTGAAGGTGGTTCTAAGCCTCGTACTTGCGATGGCAT<br>CGGGGCAGGCACTTGCTGACCTGCCAAGCAATTCCGTAGTGAGTACTGAATTTATTCTGAT<br>TCGTCTTGCTTTTGGAGCGTCTTTTTGCGTTCTATAACTGTTGTGAAAGCTACGCGGTCGC

	CATTGAAAACGAAATTAGGATTAATAAAATACCATCCTTGGCGAACATGCTTTGCAATGAT<br>TTTAGCTTTTTCTAATTCGGCTAGACCTCTTGCAAAGGTAGCTTGAGATAGTGCCAGTTTTT<br>TTTCTTGTGCGTTAAGAAAGTCCTCTAAAACGAATTTGTCTAAAGGGACGAGGTCTTTGCT<br>GATGCCTTTGTCTTGAAGTATCCAAACCAGAACGCTGAAAGCTTTTATTCCAGCGGCTCCT<br>AGTTCAAAAGTTAGCGCGATATTGGTGCTAAATAATTTTACAAATTCTTCACTATCAACACG<br>TCTGTAAGTCGTCACATGAGTGCCTTGCATCTCACCAGTGGCTTGATTGACCAGAATGTTA<br>TCATCTCGTCCTAATCGAGATAACTGAACCCTCTGACTTTTAACTGGCACAACCATACCTT<br>CGATGAAAGGATTCTCGTCATATCTGATTGGCTGCTTTCTCAATTTTGTCGCCATATTTGATA<br>AACCTTTAATCAAAAAAACCACATTTTTTGATTATACCTATTCATCGAATGAGGCAAGGTCT<br>ATCAATTTTACCCCTTTTTTTGATAGACGGTTTAATCAATATTGATAGACCCCTTCACAGATT<br>CTGAAAATCGACTTCCCTATTTTAGGGATATTTTCACGATTCCCTTTCTTAGTTCTTCCTAGT<br>GGGGAAATTCGTTGAATCCTGCCTCGGAAAAACCATGAGAAAGCTGTTGGTTATATACAC<br>GGGCAAAGCCACCCTATTTTTAGCTACTGGGGAAAGAGATAAGGCAGGTCACCAGCTCAC<br>CGTCTGAATTCGCGGCCGCTTCTAGAATTTACGATTGCTCGTCCTAAATAAATAAGAGTCTT<br>AAATATTTGTTTTAAAAATGTAATTTCAAAAATTCTCGTGATAAATTTATATGCCATAAATAT<br>AGAAAATGCAGATTTTATTTAGTATTTATAAAATATTTTAAATGTACAACAGTCTACATTCTA<br>ATAATAAAATTAGCCATTGTAGATACAGAAGATTTTCTGGTAC
**pZM3*pdc***	AGGTAGCTTGCAGTGGGCTTACATGGCGATAGCTAGACTGGGCGGTTTTATGGACAGCAA<br>GCGAACCGGAATTGCCAGCTGGGGCGCCCTCTGGTAAGGTTGGGAAGCCCTGCAAAGTA<br>AACTGGATGGCTTTCTTGCCGCCAAGGATCTGATGGCGCAGGGGATCAAGATCTGATCAA<br>GAGACAGGATGAGGATCGTTTCGCATGATTGAACAAGATGGATTGCACGCAGGTTCTCCG<br>GCCGCTTGGGTGGAGAGGCTATTCGGCTATGACTGGGCACAACAGACAATCGGCTGCTCT<br>GATGCCGCCGTGTTCCGGCTGTCAGCGCAGGGGCGCCCGGTTCTTTTTGTCAAGACCGAC<br>CTGTCCGGTGCCCTGAATGAACTGCAGGACGAGGCAGCGCGGCTATCGTGGCTGGCCAC<br>GACGGGCGTTCCTTGCGCAGCTGTGCTCGACGTTGTCACTGAAGCGGGAAGGGACTGGC<br>TGCTATTGGGCGAAGTGCCGGGGCAGGATCTCCTGTCATCTCACCTTGCTCCTGCCGAGA<br>AAGTATCCATCATGGCTGATGCAATGCGGCGGCTGCATACGCTTGATCCGGCTACCTGCCC<br>ATTCGACCACCAAGCGAAACATCGCATCGAGCGAGCACGTACTCGGATGGAAGCCGGTCT<br>TGTCGATCAGGATGATCTGGACGAAGAGCATCAGGGGCTCGCGCCAGCCGAACTGTTCGC<br>CAGGCTCAAGGCGCGCATGCCCGACGGCGAGGATCTCGTCGTGACCCATGGCGATGCCTG<br>CTTGCCGAATATCATGGTGGAAAATGGCCGCTTTTCTGGATTCATCGACTGTGGCCGGCTG<br>GGTGTGGCGGACCGCTATCAGGACATAGCGTTGGCTACCCGTGATATTGCTGAAGAGCTT<br>GGCGGCGAATGGGCTGACCGCTTCCTCGTGCTTTACGGTATCGCCGCTCCCGATTCGCAG<br>CGCATCGCCTTCTATCGCCTTCTTGACGAGTTCTTCTGAGCGGGACTCTGGGGTTCGAAAT<br>GACCGACCAAGCGACGCCCAACCTGCCATCACGAGATTTCGATTCCACCGCCGCCTTCTA<br>TGAAAGGTTGGGCTTCGGAATCGAGGATCTAGGTGAAGATCCTTTTTGATAATCTCATGAC<br>CAAAATCCCTTAACGTGAGTTTTCGTTCCACTGAGCGTCAGACCCCGTAGAAAAGATCAA<br>AGGATCTTCTTGAGATCCTTTTTTTCTGCGCGTAATCTGCTGCTTGCAAACAAAAAAACCA<br>CCGCTACCAGCGGTGGTTTGTTTGCCGGATCAAGAGCTACCAACTCTTTTTCCGAAGGTA<br>ACTGGCTTCAGCAGAGCGCAGATACCAAATACTGTCCTTCTAGTGTAGCCGTAGTTAGGCC<br>ACCACTTCAAGAACTCTGTAGCACCGCCTACATACCTCGCTCTGCTAATCCTGTTACCAGT<br>GGCTGCTGCCAGTGGCGATAAGTCGTGTCTTACCGGGTTGGACTCAAGACGATAGTTACC<br>GGATAAGGCGCAGCGGTCGGGCTGAACGGGGGGTTCGTGCACACAGCCCAGCTTGGAGC<br>GAACGACCTACACCGAACTGAGATACCTACAGCGTGAGCATTGAGAAAGCGCCACGCTT

	CCCGAAGGGAGAAAGGCGGACAGGTATCCGGTAAGCGGCAGGGTCGGAACAGGAGAGC<br>GCACGAGGGAGCTTCCAGGGGGAAACGCCTGGTATCTTTATAGTCCTGTCGGGTTTCGCC<br>ACCTCTGACTTGAGCGTCGATTTTTGTGATGCTCGTCAGGGGGGCGGAGCCTATGGAAAA<br>ACGCCAGCAACGCGGCCTTTTTACGGTTCCTGGCCTTTTGCTGGCCTTTTGCTCACATGTT<br>CTTTCCTGCGTTATCCCCTGATTCTGTGGATAACCGTATTACCGCCTTTGAGTGAGCTGATA<br>CCGCTCGCCGCAGCCGAACGACCGAGCGCAGCGAGTCAGTGAGCGAGGAAGCGGAAGA<br>GCGCCCAATACGCAAACCGCCTCTCCCCGCGCGTTGGCCGATTCATTAATGCAGCTGGCA<br>CGACAGGTTTCCCGGAAAGGTCATCCTGAAAATTCTATTCGTATTCTGATCAACCAGATCG<br>TGAATGATCGGGCGATGAGCCGGAAGCTTGTCCCGTGATCAGAACCGGCGCAGCCTCTTC<br>CCTTTTATCTATTTCAAATTCAGGCCTGCCACAGCGGAGAATCTTCGCTGTGGCTTTTTTGC<br>GTCCAAGACCGGAGCCCGCACAATGACCGCCTCTTCCTCTTCAGCGCCCCTCTCGGATGC<br>CTCTGTCTTTTCTTTAGAGCCTGTCTCTGCGTCTTTTGAACAAACGACGCCCGTAACCAGT<br>CCTTTTATCGACAGGCTAGGCCGTTTCAAGCGTGGCACTTATCGAACCATGTTGGTTGAAA<br>CGGGTATGCCATATGACAGCGCAAAGATTATGGCAGATCAGGCCGCAGAACAACATAAAA<br>GAGAGCGCACATTCTCTAAATTAATGCCCTTGGCCAATGGCGCTCATCCCGAAATTTCGGC<br>CTCAAATACCAATATTGAGGCTACAGCCTCTGCATCAGATTTCGACGAAATCGAATATTTTG<br>ACCAGATGGTATCAAACGGCGTTGATCCTAGAGATGCCGCAGATATTGCTCGCAAAACAG<br>CTATTGCGAGACGTCGAGACCCTCTCGAACGAGCCATTCGCTATAAACCCGCCGTCAAA<br>CTCCTGATCCTGTGCATCGGGATTCTTATGAGGTGGGCGAACGCGAGAAGTCGGTCTGGA<br>AGCCGGTCAATCCGCAAGAGATTGGTGCCTATCTGGAAGCGGTTGATCAATATTCGATCAA<br>AACCAAGGGCTTAAGCGATAAGGCCGTTCGTCTTCTAAAGATGCTGTTTCGGATGGTCGA<br>CTTTAAAACTGGCCGTCTGGAGCCGACCCTCGACACAATATGCGATCGTGTCGGTTATGCC<br>CGTGCGACGGTCGTCAAATTACTGCGGCAATTACAGGATTTAGGCTTCATCCGCTGGATAC<br>GCCGGTCAATCAAGATCAAGGCGGACGGCAAAGGCCCCCGCCGCAAGCAGACCAGTAAC<br>GCCTACGGCTTCTTGTCTCCCAAGGCTTGGCCAGATTTGGCCAGACAGGTTTTCGAGCGT<br>GTGATGCGCCGGAGGCATGCGCCTATTCCTGACGATATCGACCACGCCCAAGAGGCGGAC<br>AAAGCCGAAACCAAGGCTATCGTTGAAAGCTTGCCGCCCGAAGATTTCGTCAAAGCAATA<br>TCGGGCGATAAAGAGCCAACAGAGTTAACAGATTCGTTAATACAGCTCGCCAAATCTATC<br>GAGCGGGCGGAACAACGGGAACAAGAAGAACAGGCTAAGAAGGCAGCACAGGCCGGT<br>CAGGAAGAGGTTAAAACATCTTCCCAAACACCTAAGGAGGATACCTCAAAAAAGCCCCC<br>AATCTCGGGCAATCAACGCGAGTTCAATTCTTATACTCTATCCTGTTATACTTATTTATCTAA<br>TAGCGACCATACAAAACTAAAAACCAAAGAAGAAGATAAAGACTGCACTGCTCCTGCTA<br>CCGCAGGCAGTGCAGACCCTATTTATGATCGAAAACCTGACAGCAATCCTTTCCTCTCAAC<br>TCTCAAAGCTCGCGGTTATCGTCCTGATCAAATCAACGCTTGCCTCGAAAACAGAGATAA<br>ACGGAAAAAGAAAGGATGGATGCCTCTGTCACCCGATGAAACCGCCCGCCTCCTTATTCA<br>AACAAAACAGATGTGCCCATAACAACGACCACTATCCTTCTCAAAGCATATCGTCCGTTAA<br>TCTCTCGGCGCTGCTCGCTTCGCTCACAGGCTCGGCGAGAAAGAAATTTGCCTTTCGGGG<br>GACTATCTAATTCCTAACAATTCGCATTATCGATAATGTAATAAAAGGCTAATCTTTAAAAA<br>CCAAATTACAGCGATTACATCATTTCTATGAATGTGTATTATACATAATATGAGAAGTCGTGA<br>TTTGATCAAAGAAGTTAAAAAAGCCAATATTACCAGCTTGACAGATATAAAATAGGCCTTG<br>GTTGATTTCGTGAAAGAACTGGAAGATTGCACAATTCTTCTTTCTGGTGATATCTTTGACC<br>CGACGGCCCTCAGTCAATGAGTGTGCGCCGCATTTCTGCCCATTCAATCAGGGCCTGCATT<br>GAAGGGCCGAGAGCGTATCCCATACCGCTGAGCTCGTATTCTACCTTTGGCGGGACCTGC<br>GGATAGACCGTTCGGAGCACGATGCGATCCTTTTCCAGTTGCTTGAGCTGCTGGATCAGCA

	TCTTCTGGTTGATGCCGTCGATCTGCTTTTCCAGTGAGGAGAAGCGGATTGGGCCTTTTGC<br>GGCAAACAACTGGCAGATGATAACAATCTTCCATTTTCCTTCGAGTACCCTGAGCGCTTCT<br>GTCGTCGCCTCAGTTACGCTCATGATCGCGGCATGTTCTGATATTTTTCCTCTAAAAAAGAT<br>AAAAAGTCTTTTCGCTTCGGCAGAAGAGGTTCATCATGAACAAAAATTCGGCATTTTTAA<br>AAATGCCTATAGCTAAATCCGGAACGACACTTTAGAGGTTTCTGGGTCATCCTGATTCAGA<br>CATAGTGTTTTGAATATATGGAGTAAGCAATGAGCAAAGGAGAAGAACTTTTCACTGGAG<br>TTGTCCCAATTCTTGTTGAATTAGATGGTGATGTTAATGGGCACAAATTTTCTGTCAGTGG<br>AGAGGGTGAAGGTGATGCTACATACGGAAAACTCACCCTTAAATTTATTTGCACTACTGGA<br>AAACTACCTGTTCCATGGCCAACACTTGTCACTACTCTGACCTATGGTGTTCAATGCTTTT<br>CCCGTTATCCGGATCACATGAAACGGCATGACTTTTTCAAGAGTGCCATGCCCGAAGGTTA<br>TGTACAGGAACGCACTATATCTTTCAAAGATGACGGGAACTACAAGACGCGTGCTGAAGT<br>CAAGTTTGAAGGTGATACCCTTGTTAATCGTATCGAGTTAAAGGGTATTGATTTTAAAGAA<br>GATGGAAACATTCTCGGACACAAACTAGAGTACAACTATAACTCACACAATGTATACATCA<br>CGGCAGACAAACAAAAGAATGGAATCAAAGCTAACTTCAAAATTCGCCACAACATTGAA<br>GATGGTTCCGTTCAACTAGCAGACCATTATCAACAAAATACTCCAATTGGCGATGGCCCTG<br>TCCTTTTACCAGACAACCATTACCTGTCGACACAATCTGCCCTTTCGAAAGATCCCAACGA<br>AAAGCGTGACCACATGGTCCTTCTTGAGTTTGTAACTGCTGCTGGGATTACACATGGCATG<br>GATGAGCTCTACAAA

Supplementary Table S3. Primer sequence for qPCR of the main genes involved in ED pathway

**Primers**	**Sequences (5'- 3')**
16sRNA-F	TCACCGCCATTGTAGCAC
16sRNA-R	AGTTGGGCACTTTAGAGGAAC
ZMO0293-F	AAGCATTCAGACCGCCATA
ZMO0293-R	TGCGGGTGGTATTCAGGTTA
ZMO0366-F	GGTTGGTGATGAAGGGACGT
ZMO0366-R	TTTGTAAAGCCGAACCACCG
ZMOO152-F	CAGACCAAACCAAGACGACG
ZMO0152-R	CTGAAAACGCCAGCAAAACC
ZMO0177-F	ATGACCTGGGTAGCGTTGAA
ZMO0177-R	TCACGATATCAACGCCAAGC
ZMO0178-F	ATTTTGGCTCACTTCGGTCG
ZMO0178-R	TGATGAAGTGAACCGGACGA
ZMO0367-F	TGGTTTCCGTGATTTTGCAGA
ZMO0367-R	AGGTCAGCTAATTTTCCGAATTG
ZMO0368-F	CCGTCCAAACCTGTCCTGTA
ZMO0368-R	GCGAGCAAATACTTTCATCTGC
ZMO0369-F	CGACTTTTAAAACGGCAGAACA
ZMO0369-R	CGTATCGATGTCCAGCTTTTCA
ZMO0487-F	AGACAAAGAGCAAGCCAGAG
ZMO0487-R	AAGAAGTCAAAGCAGCACCG
ZMOO569-F	GACCCCTATAATGCGGCTGA
ZMO0569-R	CCAGTCCAGAAAATTATGCGC
ZMO0997-F	TGGTGGTCTGAACGTTCTTG
ZMO0997-R	GTCAGACCCGGGCTAACGAA
ZMO01236-F	CGATACCGTCAAGCACCAAG
ZMO1236-R	CGGTGGTGACAGCTGTTGCC

ZMO1237-F	GCAAAGACGCTCCCGATATG
ZMO1237-R	GAATCGGCAAATTTCACGCG
ZMO1240-F	CTTGCCAGCCTTCAGTTCAG
ZMO1240-R	CAAGTTCGATCTGTCTGGCG
ZMO1360-F	TCTTGATGTTGTTGTACGGACC
ZM01360-R	TCAGGAAGTCGCTCAGATGG
ZMO1478-F	GACAAGCTAAGGGTTACGCG
ZMO1478-R	TTCCCAGGTCCCAATTTCGA
ZMO1496-F	CGGGGTATTGTTGACAGCAC
ZMO1496-R	TCAGGATCAAGGGCATCTCC
ZMO1570-R	CGATTTGAAAGGCCCCGTTG
ZMO1570-F	GCCCCTGCCCCGAAATAACC
ZMO1596-F	AATCCGGTGTTGTGAAGCAG
ZMO1596-R	CACCGAGGGAGATGACGAAG
ZMO1608-F	GGCTACCGGTCTTGATCTGA
ZMO1608-R	GCTGCTCGTGAAGTTTAACCA
ZMO1719-F	CCACAACCCCTGAAGAAACG
ZMO1719-R	GTTTCGGGGTGTTGGTGATA
ZMO1722-F	AAGCAGAGCCTTTCCAAACG
ZMO1722-R	CTTGGAAGCCTGTCATCGTG
ZMO1754-F	ACCGCATAAATCTGGGCAAT
ZMO1754-R	CGGTTCAAAATGGCGCAAAG
ZMO1955-F	CCTTTATTCTGTGCTGCTGGT
ZMO1955-R	ACGCCTATATTTTCCCGGGT
ZMO1963-F	GCGGTAAACATAGAAGTCGGG
ZM01963-R	CTCGCGCCTTGGAAGAAATC
